# Development and Psychometric Properties of Coping Scales towards Adherence to Pharmacological Treatment, Heart-healthy Eating and Cardiovascular Physical Exercise

**DOI:** 10.11621/pir.2024.0307

**Published:** 2024-09-15

**Authors:** Jessica Berenice Flores-Mendoza, Mirna García Méndez, Andrea Bravo Doddoli, Rolando Díaz-Loving

**Affiliations:** a *Faculty of Higher Studies Zaragoza, National Autonomous University of Mexico, Mexico*; b *Faculty of Psychology, National Autonomous University of Mexico, Mexico*

**Keywords:** coping, psychometrics, therapeutic adherence, medication, heart-healthy eating, cardiovascular psysical exercise

## Abstract

**Background.:**

Coping, within Lazarus transactional theoretical framework, is conceptualized as a dynamic set of cognitive and behavioral processes that adapt continually to manage specific demands, whether internal or external, perceived as exceeding an individual’s available resources. This framework supports understanding healthy coping behaviors, especially regarding adherence to treatment in cardiovascular disease management.

**Objective.:**

Develop and validate coping scales designed to assess adherence to pharmacological treatment, heart-healthy eating, and cardiovascular physical exercise in mexican patients diagnosed with ischemic heart disease.

**Design.:**

The research employed both qualitative (focus groups) and quantitative (exploratory and confirmatory factor analysis) methodologies to ensure robustness. The coping scales underwent rigorous testing, including exploratory factor analysis (EFA) and confirmatory factor analysis (CFA), which revealed a two-factor solution for medication adherence coping, a three factors solution for physical exercise adherence coping, and a three factors solution for healthy eating adherence coping.

**Results.:**

The findings suggest that coping strategies, although universal in nature, are sensitive to cultural nuances among patients with ischemic heart disease. By capturing the complexities of coping behaviors within a specific cultural context, these scales provide valuable insights into the multifaceted nature of adherence to primary prevention measures.

**Conclusion.:**

This study contributes to the understanding of how coping mechanisms intersect with cultural factors in the management of chronic conditions such as ischemic heart disease, highlighting the importance of tailored interventions that consider patients cultural backgrounds and individual coping styles.

## Introduction

Coping, within Lazarus’ (2000) transactional theoretical framework, is conceptualized as a dynamic set of cognitive and behavioral processes that are continuously adapted to manage internal or external demands perceived as exceeding an individual’s available resources. It emphasizes not only the importance of having personal resources but also the ability to apply them effectively in response to those demands. Sandín and Chorot (2003) suggest that coping styles help explain differences in stress adaptation, while Zavala et al. (2008) define coping as cognitive and behavioral strategies employed to manage everyday stress, ranging from avoidant approaches to positive reappraisal strategies.

Coping styles generally fall into two categories: *problem-centered coping*, aimed at modifying the environment to resolve stress, and *emotion-centered coping*, focused on regulating emotions related to stress (Lazarus & Folkman, 1984; Folkman et al., 1987). Moos and Shaefer (1986) add a third style, *problem-assessment-centered coping*, which adjusts the perception of a stressful situation through logical analysis and cognitive avoidance. The coping function is closely linked to decision-making, information seeking, maintaining autonomy, and the capacity to direct resources to meet stressful demands (Billings & Moos, 1984). Morris (1997) found that in patients with ischemic heart disease, passive coping increased diastolic blood pressure, while active coping elevated heart rate and systolic pressure, potentially contributing to cardiovascular issues.

Silva and Agudelo (2011) analyzed beliefs about disease and coping strategies as predictors of health-related quality of life in cardiovascular patients. Their findings indicated that passive coping strategies and disease-focused beliefs were linked to low quality of life, while religion served as a protective factor. Similarly, Casagrande et al. (2019) found that individuals with hypertension and heart disease used fewer problem-focused coping strategies, and Castillo et al. (2019) observed that obese adolescents employed unproductive coping strategies despite occasional efforts toward problem-solving. Depression and anxiety were associated with higher cardiovascular risk. These findings highlight the importance of understanding coping styles in clinical contexts, especially for chronic diseases like cardiovascular conditions.

Coping styles have been assessed through various methods, including semistructured interviews, self-administered questionnaires, and inventories. Billings et al. (2000) used an interview based on the Folkman and Lazarus (1988) Coping Styles Scale, though it lacked strong reliability and validity. Other tools, such as the COPE Inventory (Carver et al., 1989) and the Ways of Coping Instrument (WCI) by Lazarus and Folkman (1984), have been more widely used and validated. These instruments are grounded in the Lazarus and Folkman model, focusing on different coping strategies, including problem-solving and emotional coping.

The WCI assessment tool evaluates coping capacity by asking individuals to recall recent stressful situations, distinguishing between problem-focused and emotion-focused strategies. The COPE Inventory evaluates stress responses through problem-focused and emotion-oriented scales, while Amirkhan’s (1994) Coping Strategy Indicator (CSI) has shown consistent psychometric results in various settings. Other scales, like Ryan-Wenger’s Schoolagers’ Coping Strategies Inventory (SCSI), offer age-specific methodologies for assessing coping capacities within children.

Endler and Parker’s (1990) Multidimensional Coping Inventory (MCI) assesses task-oriented, emotion-oriented, and avoidance-oriented coping styles, demonstrating high validity and correlations with depression, anxiety, and personality measures, particularly in collectivistic cultures. Fernández-Abascal’s (1997) Coping Styles and Strategies scale, known for its high internal consistency, has been used in Spanish and Argentinian samples. Similarly Chorot and Sandín’s (1993) Revised Coping Strategy Scale (EECR), a modified version of the WCI, has been widely applied by Colombian psychologists. Other scales, like the Health and Daily Life Scale and the Multidimensional Scale of Coping Styles by Góngora and Reyes (1998), exist but have not been validated for the Mexican population.

Ischemic heart disease is one of the leading causes of mortality both in Mexico and globally. Current assessments lack consideration for how patients with this condition manage adherence to a comprehensive regimen encompassing medication, a heart-healthy diet, and physical exercise. This research aims to design and validate scales for evaluating coping styles related to these specific health behaviors, providing valuable tools for clinical settings. Such scales will enable healthcare providers a means to better understand and support patients in managing their condition effectively. However, the literature review reveals an absence of instruments specifically designed to evaluate coping styles focused onadherence to comprehensive treatments (including medication, heart-healthy diet, and cardiovascular physical exercise) for ischemic heart disease, one of the most prevalent diseases in Mexico and the world. Therefore, this study aimed to design and validate three scales assessing coping styles specifically oriented toward these health behaviors, to facilitate application in hospital environments and enable characterization of coping strategies among patients with cardiovascular disease.

## Methods

### Participants

Patients from a tertiary care public health hospital who were over 18 years of age and had a diagnosis of ischemic heart disease based on the criteria of the New York Heart Association (NYHA) (McMurray et al., 2012) participated. Those who had a neurological disorder or psychiatric or intellectual disability were excluded.

As it was a non-experimental cross-sectional study, there were no losses of participants in any of the phases, since all of them agreed to be part of the evaluation carried out. At the end of each phase, participants were given a certificate of attendance with curricular value, recognizing their contribution and encouraging full engagement.

#### Sample 1 (Focus groups)

Two focus groups were assembled with 4 participants each. The sample had an age range of 38 to 80 years (M = 55 years, SD = 14.6), most indicated that they were men (60%) and the rest women (40%). Regarding marital status, all reported being married. 40% of the participants indicated they were active professionals, 20% were retired, 20% were merchants, and 20% of the participants indicated that they were housewives. Finally, 65% indicated that they were religious believers and 35% atheists.

#### Sample 2 (Exploratory Factor Analysis)

A total of 277 patients with an age range between 32 and 82 years (M = 56 years, SD = 9.6) participated, 44% were women and 56% were men. Regarding marital status, 53.6% reported being married, 31.2% being single, 7.2% in a common-law union, 4% widowed and 4% separated. In relation to their occupation, 45% indicated that they were professionals who worked in a public or private institution, 27% indicated that they were retired, 15% were engaged in commerce and 13% indicated that they were housewives. Finally, 75% were religious believers and 25% were atheists.

#### Sample 3 (Confirmatory factor analysis)

A total of 317 patients between 30 and 82 years of age (M = 52 years, SD = 12.15) participated, of which 43% were women and 57% were men. Regarding marital status, 53.4% reported being married, 28.2% single, 8% in a common-law union, and 5.2% widowed, and 5.2% separated. In relation to their occupation, 13% indicated that they were housewives, 13% were engaged in commerce, 29% were retirees and 45% were professionals who worked in a public or private institution. Finally, 72% were religious believers and 28% were atheists.

### Procedure

Prospective participants were recruited through invitations at a third level carepublic health hospital in Mexico City. Data collection was conducted using the online platform Google Forms. Upon accessing the form, participants encountered an informed consent that ensured the anonymity and confidentiality of their data, which had been previously approved by the local Research, Ethics and Biosafety committee of the hospital with registration number 463.2020. The research was carried out in accordance with the ethical guidelines expressed in the Declaration of Helsinki of 1975 and the articles of the psychologist’s code of ethics: Articles 8, 9, 12, 15, 16, 17, 18 and 49 for research on human subjects (Mexican Society of Psychology, 2007).

Exploratory factor analysis (EFA) was performed following the recommendations of Lloret-Segura et al. (2014). First, the correlation matrix was assessed for its suitability for factor analysis by evaluating the Kaiser-Meyer-Olkin (KMO) and Bartlett sphericity indices. Additionally, the criteria used to decide how many factors to retain included Kaiser’s rule (i.e., eigenvalues greater than 1) and the interpretability of the solution obtained. Finally, the reliability of the scales were evaluated using Cronbach’s alpha (Ventura-León et al., 2017).

Confirmatory Factor Analysis (CFA) was performed using the structural equation modeling (SEM) approach (Kline, 2016). Model fit was assessed following the recommendations of Marsh et al. (2004), i.e., CFI and TLI ≥ .90 and RMSEA and SRMR ≤.08.

## Results

In accordance with the guidelines proposed by Lloret-Segura et al. (2014) for assessing adherence to pharmacological treatment using the coping scale, a factor analysis was carried out using the unweighted least squares method with oblimin rotation applied. Those items that indicated communalities exceeding .30 and factorial loads equal to or greater than .40, and presented eigenvalues greater than 1, were retained. Following the application of this criterion, 2 factors called *positive reevaluation* (α = .85) and *evasion* (α = .81) were formed, with 12 items that explain 57.12% of the accumulated variance, with a global Cronbach’s alpha of .84. The results are shown in *Table 1*.

**Table 1:** The factorial and psychometric structure of the Medication Adherence Coping Inventory

	1	2	Total
Items	6	6	12
Factorial variance	29.41	27.70	57.12
Cronbach’s Alpha	.855	.815	.846
1. Me inspire a generar un calendario con fechas y horas específicas para la toma de mis medicamentos (I was inspired to create a calendar with specific dates and times for taking my medications)	.830		
2. Intente sentirme mejor tomando mis medicamentos para el corazón (I tried to feel better by taking my heart medications)	.827		
3. Soñé o imagine que las cosas eran mejores cuando tomaba mis medicamentos (I dreamed or imagined that things were better when I took my medications)	.791		
4. Busqué un poco de esperanza intentando mirar los beneficios de la medicación (I sought a glimmer of hope by trying to focus on the benefits of medication)	.780		
5. Cambié y madure como persona al tomar mis medicamentos a diario y a tiempo (I changed and matured as a person by taking my medications daily and on time)	.643		
6. Hice algo para compensar el hecho de que no había tomado mis medicamentos tal como lo indicó mi medico (I did something to make up for not taking my medications as my doctor instructed)	.515		
7. Me di cuenta de que yo mismo no quiero tomar mis medicamentos (I realized that I myself don’t want to take my medications)		.811	
8. Evité que los demás se enteraran de que no estoy tomando mis medicamentos tal como me lo indicó mi médico (I prevented others from finding out that I’m not taking my medications as my doctor instructed)		.769	
9. Espere a que ocurriera un milagro para que tomara cada uno de los medicamentos prescritos por mi médico (I waited for a miracle to happen so that I would take each of the medications prescribed by my doctor)		.727	
10. Me negué a creer que no estaba tomando mis medicamentos (I'refused to believe that I wasn’t taking my medications)		.699	
11. Espere a ver que pasaba antes de tomar los medicamentos para mi corazón (“I waited to see what would happen before taking the medications for my heart”)		.695	
12. Seguí adelante con mi destino (simplemente algunas veces tengo mala suerte para recordar tomar mis medicamentos) (I'carried on with my fate (sometimes I just have bad luck remembering to take my medications))		.535	

To perform the EFA, an assessment of the multivariate normality of the data was conducted. This was done by calculating the multivariate kurtosis coefficient using Mardia’s test. The value obtained was 60.83, which fell below the limit established by Bollen (1989), where the limit for 20 observed items corresponds to 440. Once this evaluation was completed, the adjustment of the model obtained in the exploratory EFA was examined.

**Figure 1.**
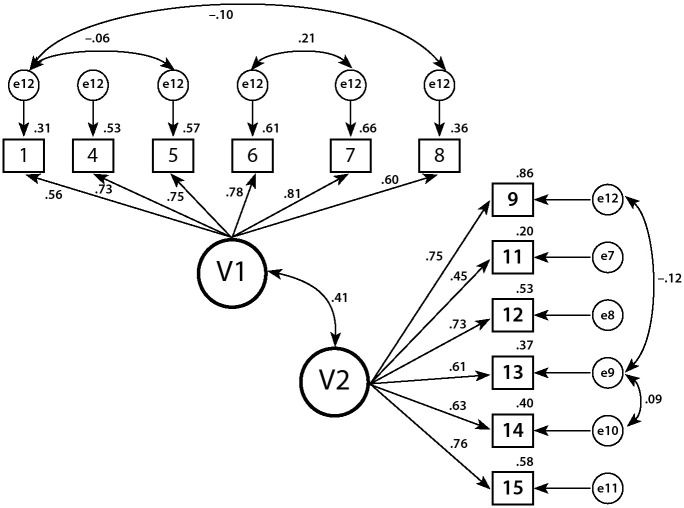
Confirmatory Factor Analysis (CFA) of the Coping Scale model for Adherence to Cardiovascular Pharmacological Treatment

The resulting model is presented in *Figure 1*, where the standardized factor coefficients are shown along with the adjustment indices obtained. Although the Chi-square test did not indicate an optimal fit, the other fit indices revealed satisfactory results.

### Coping Scale for Adherence to Cardiovascular Physical Exercise

Following the recommendations of Lloret-Segura et al. (2014), a factor analysis was performed using the unweighted least squares estimator with oblimin rotation for the scale assessing coping strategies related to adherence to physical exercise. Items with commonalities greater than .30 and factor weights greater than or equal to .40 were retained, resulting in a criterion that grouped 15 items into three factors with eigenvalues greater than 1. These factors were named: *positive reevaluation* (α = .811), *evasion* (α = .704) and *reflexive cognitive analysis* (α = .703). Together these factors explained 44.83% of the accumulated variance, with an overall Cronbach’s alpha of .84, indicating good reliability. The results are shown in *Table 2*.

**Table 2:** The factorial and psychometric structure of the Coping Scale for Adherence to Cardiovascular Physical Exercise

	1	2	3	Total
Ítems	6	4	5	15
Factorial Variance	19.10	13.44	12.281	44.83
Cronbach’s Alpha	.811	.704	.703	.844
1. Intenté sentirme mejor haciendo caminatas más de 30 minutos al día (I tried to feel better by taking walks for more than 30 minutes a day)	.754			
2. Busqué un poco de esperanza intentando mirar las cosas buenas del ejercicio físico cardiovascular (I sought a bit of hope by trying to see the good things about cardiovascular physical exercise)	.728			
3. Me consolé pensando que las cosas podrían ser mejores si realizo mi ejercicio físico (I consoled myself by thinking that things could be better if I do my physical exercise)	.659			
4. Tuve fe en hacer el ejercicio que me corresponde (I had faith in doing the exercise that is required of me)	.611			
5. Me inspire a hacer ejercicio físico de manera creativa (I was inspired to exercise creatively)	.578			
6. Cambie y maduré como persona al caminar por 30 minutos al día de manera frecuente (I changed and matured as a person by frequently walking for 30 minutes a day)	.528			
7. Me di cuenta de que yo mismo no quiero realizar ejercicio físico (I realized that I myself don’t want to engage in physical exercise)		.780		
8. Me negué a creer que no estaba realizando ejercicio físico (I'refused to believe that I wasn’t engaging in physical exercise)		.643		
9. Evité que los demás se enteraran de que no estaba realizando ejercicio físico (I avoided letting others know that I wasn’t engaging in physical exercise)		.580		
10. Trate de olvidarme por completo de que no estoy realizando ejercicio físico (I tried to completely forget that I’m not engaging in physical exercise)		.518		
11. Tuve el deseo de que mi flojera por hacer ejercicio físico terminara (I had the desire for my laziness regarding physical exercise to end)			.669	
12. Imagine el modo en que puedo hacer ejercicio físico (I imagined how I could exercise)			.642	
13. Hice algo para compensar mi falta de ejercicio físico (I did something to compensate for my lack of physical exercise)			.632	
14. Seguí realizando ejercicio físico al ver los beneficios de este (I'continued to exercise upon seeing the benefits of it)			.443	
15. Analice diversas formas para realizar ejercicio físico cardiovascular (I analyzed various ways to engage in cardiovascular physical exercise)			.414	

To perform the Confirmatory Factor Analysis (CFA), the multivariate normality of the data was first assessed using the Mardia test, which yielded a multivariate kurtosis coefficient of 33.006. This value is below the threshold set by Bollen (1989), which indicates that for 15 observed items the limit would be calculated as15 (15+2) = 255. Once this test was performed, the fit of the model obtained in the EFA was evaluated.

**Figure 2.**
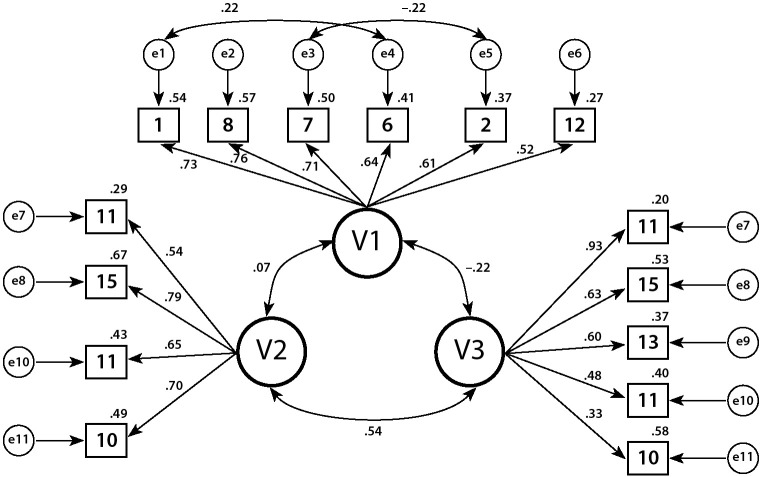
Confirmatory Factor Analysis (CFA) of the Coping Scale for Adherence to Cardiovascular Physical Exercise model

The resulting model is shown in *Figure 2*, where the standardized factor coefficients can be seen with the adjustment indices obtained. With respect to Chi-square, it did not have a good fit, the other adjustment indices showed satisfactory results (Hu & Bentler, 1999; Kline, 2005). The adjustment indices were: χ^2^ (68) = 194.38; CMIN/DF=2,859; TLI =.903 NFI=.911; IFI=.921, CFI=.925, SRMR= .0487; RM-SEA=.051(.043–.060).

### Coping Scale for Adherence to Heart-Healthy Eating

Following the guidelines established by Lloret-Segura et al. (2014) for the assessment of adherence to physical exercise through the coping scale, a factor analysis was carried out using the unweighted least squares method along with oblimin rotation. Items sharing commonalities greater than .30 and factorial loads equal to or greater than .40 were retained, alongside those presenting eigenvalues greater than 1. According to these criteria, 3 factors were identified: reflective cognitive analysis (α = .76), negative self-focus (α = .77) and avoidance (α = .69), made up of 12 items, which explained 63.12% of the accumulated variance, with a global Cronbach’s alpha coefficient of .78. The results are detailed in *Table 3*.

**Table 3:** The factorial and psychometric structure of the Coping Scale for Adherence to Heart-Healthy Eating

	1	2	3	Total
Items	5	3	4	12
Factorial variance	32.85	20.72	9.54	63.12
Cronbach’s Alpha	.763	.771	.698	.785
1. Intente sentirme mejor comiendo y bebiendo lo que me corresponde (I tried to feel better by eating and drinking what I should)	.766			
2. Soñé o imagine que las cosas eran mejores cuando hacia mi dieta cardiosaludable (I dreamed or imagined that things were better when I followed my heart-healthy diet)	.751			
3. Busqué un poco de esperanza intentando mirar las cosas buenas de la dieta cardiosaludable (I sought a glimmer of hope by trying to focus on the benefits of a heart-healthy diet)	.749			
4. Me inspire a hacer comidas sanas de manera creativa (I was inspired to creatively make healthy meals)	.738			
5. Me dije cosas que me ayudaron a sentirme mejor para hacer la dieta prescrita por mi médico (I told myself things that helped me feel better about following the diet prescribed by my doctor)	.522			
6. Esperé a que ocurriera un milagro para que hiciera la dieta prescrita por mi médico (I waited for a miracle to happen so that I would follow the diet prescribed by my doctor)		.881		
7. Seguí adelante con mi destino (simplemente algunas veces tengo mala suerte para hacer la dieta cardiosaludable) (I carried on with my fate (sometimes I just have bad luck following the heart-healthy diet))		.782		
8. Me disculpe o hice algo para compensar el hecho de que no había realizado la dieta prescrita por mi médico (I'apologized or did something to compensate for not following the diet prescribed by my doctor)		.737		
9. Trate de olvidarme por completo de que no estoy realizando la dieta cardiosaludable (I tried to completely forget that I’m not following the heart-healthy diet)			.786	
10. Evite que los demás se enteraran de que no hago la dieta prescrita por mi médico (I prevented others from finding out that I’m not following the diet prescribed by my doctor)			.755	
11. Me negué a creer que no estaba realizando la dieta cardiosaludable (I refused to believe that I wasn’t following the heart-healthy diet)			.536	
12. Me di cuenta de que yo mismo (a) no quiero hacer la dieta cardiosaludable (I realized that I myself don’t want to follow the heart-healthy diet)			.356	

Before conducting the AFC Confirmatory Factor Analysis, an assessment of the multivariate normality of the data was carried out. This was done by calculating the multivariate kurtosis coefficient using Mardia’s test. The value obtained was 60.83, which was below the limit established by Bollen (1989), which for 12 observed items would correspond to 168. Once this evaluation was completed, the adjustment of the model obtained in the EFA was examined.

**Figure 3.**
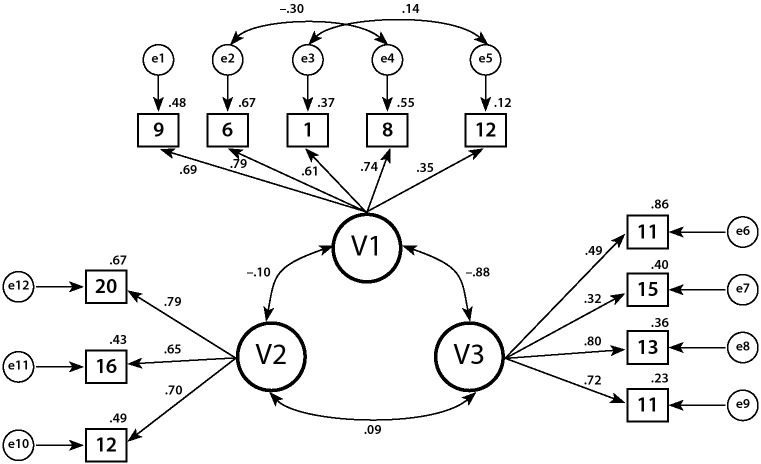
Confirmatory Factor Analysis (CFA) of the Coping Scale for Adherence to Heart-Healthy Eating

The model is presented in *Figure 3*, where the standardized factor coefficients are shown along with the adjustment indices obtained. Although the Chi-square test did not show a good fit, the other adjustment indices showed satisfactory results (Hu & Bentler, 1999; Kline, 2005). The adjustment indices were: x2(48) = 162.603; CMIN/DF = 3,387; TLI = .903 NFI = .906; IFI = .9361, CFI = .934, SRMR = .0487; RM-SEA=.071(.043–.060).

## Discussion

The objective of this study was to develop and establish the psychometric properties of measurement instruments to evaluate coping with adherence to primary prevention measures in a sample of patients diagnosed with ischemic heart disease. To achieve this objective, three scales were generated from a qualitative and quantitative study in accordance with the literature. As a result, it is possible to assert that the developed scales of coping with medication adherence, heart-healthy eating and cardiovascular physical exercise evaluate universal elements of these constructs while simultaneously being sensitive to relevant elements of Mexican culture.

With reference to the factors resulting from the scale of coping with adherence to pharmacological treatment, two were found and confirmed. The first of these factors, termed positive reevaluation, involves the reinterpretation of the challenges associated with treatment in a more optimistic and constructive way. People who employ this coping style seek to find positive aspects within the situation, such as the long-term benefits of treatment, social support, or the personal growth that may emerge from the experience (Moos & Shaefer, 1986). Rather than focusing solely on the difficulties and limitations of drug treatment, those who use positive reappraisal seek to change their perspective to reduce stress and promote a more proactive attitude towards treatment adherence (Zavala et al., 2008). This approach can help improve motivation and perseverance, which in turn can lead to greater adherence to their medication prescription, and ultimately better health outcomes (Cando & Bermeo, 2016). The second resulting factor, termed, avoidance, refers to the tendency observed in some people to avoid or evade responsibility to adhere to the prescribed treatment. Those who exhibit this coping style may experience anxiety, fear, or discomfort with medication, leading them to avoid taking medications as directed (Mikulic & Crespi, 2008). Instead of actively facing the challenges associated with treatment, they choose to avoid the situation altogether, which can result in non-adherence and negative health consequences.

In relation to the factors of the scale of coping with adherence to cardiovascular physical exercise, three were found. The first factor, termed avoidance, reflects the desires and behavioral efforts to escape or evade cardiovascular physical exercise. In this context, those who exhibit avoidance may experience anxiety, fear, or discomfort at the thought of cardiovascular physical exercise. Instead of actively addressing these emotions and seeking solutions to overcome the barriers they face, they instead choose to avoid physical activity altogether (Endler & Parker, 1990, Carver, 1997). This coping style can manifest itself in a variety of ways, such as avoiding environments where exercise is performed, constantly delaying the start of an exercise routine, or finding excuses not to participate in planned physical activities (Patten et al., 2021). Although avoidance can provide temporary relief from perceived discomfort, in the long term it can have negative consequences for physical and mental health, as it limits opportunities to maintain an active and healthy lifestyle (Del Castillo et al., 2013). The second factor of the scale, called positive reappraisal, reflects the efforts made by individuals to make meaning from the performance of cardiovascular physical exercise and its perception as positive personal growth. Finally, the third factor called reflective cognitive analysis, involves the process of self-reflection exercises that help individuals analyze and problem solve (Lazarus, 2006; Folkman, 2010).

Reflexive cognitive analysis, as a coping style in cardiovascular physical exercise, involves a conscious and introspective approach to physical activity. People who employ this coping style tend to reflect deeply on their participation in cardiovascular exercise, carefully considering their motivations, beliefs, and perceptions associated with this activity (Góngora & Reyes, 1998). Rather than simply reacting to perceived stress or discomfort during exercise, those who use reflective cognitive analysis strive to understand the underlying causes of their emotions and thoughts (Urbano-Reaño, 2022). This may involve critically examining their expectations, identifying potential obstacles, and developing strategies to deal with challenges effectively. In addition, reflective cognitive analysis in cardiovascular physical exercise may include the application of positive thinking and cognitive restructuring techniques to promote more adaptive attitudes toward physical activity (Figueroa López et al., 2017). Ultimately, this coping style fosters greater awareness and self-efficacy in relation to exercise, which may consequently contribute to more consistent and rewarding participation in cardiovascular physical activities (Carver et al., 1989).

As for the factors derived from the coping scale of adherence to heart-healthy eating, three resulted were obtained. The first of these was avoidance, reflecting the degree of desire and behavioral effort to escape or evade the consumption of heart-healthy foods. The second was the presence of a negative self-focus, indicative of a perceived inability to cope with a given situation and a belief that things usually go wrong. The third factor, called reflective cognitive analysis, indicated a conscious, thoughtful approach to food choices. In this context, people who employ this coping style tend to reflect on their food choices and carefully consider nutritional aspects and the effects on their health (Moos & Shaefer, 1986). This involves evaluating food choices from an informed and rational perspective, considering how each choice contributes to your long-term physical and mental well-being. In addition, this coping style may involve actively seeking nutrition information and adopting healthy eating habits as a strategy to cope with stress and promote a balanced lifestyle (Dawson & Golijani-Moghaddam, 2020). The second factor, called negative self-focus, refers to a perceived inability to cope with a given situation and a belief in an inability to eat in a heart-healthy way. The third factor, called reflective cognitive analysis, involves a conscious and reflective approach to food decisions including a consideration of the nutritional aspects and effects particular foods may have on cardiovascular health (Soria-Trujano et al., 2009; Valdez et al., 2022). According to this approach individuals reflect on their eating habits, identify emotional or situational triggers, and develop strategies for adopting a healthier diet. Additionally, reflective cognitive analysis is observed to foster greater awareness and self-efficacy in eating, thus promoting a more balanced and heart-friendly diet (Zavala et al., 2008).

## Conclusion

In this paper, three scales are presented that have demonstrated their validity and reliability according to the guidelines proposed by Reyes-Lagunes and García and Barragán (2008) and the guidelines outlined by Lloret-Segura et al. (2014) and are consistent with the theoretical proposal of Lazarus and Folkman (1986). From the point of view of their practical application, these scales can be utilized to characterize a specific population in studies aimed at measuring coping in adherence to primary prevention measures in DKA. They can also serve as indicators to guide the development of strategies, programs and interventions, as well as evaluating the impact on coping as an intermediate outcome (Flores-Mendoza et al., 2022).

## Limitations

Although three scales were developed and validated to assess coping with adherence to primary prevention measures in patients with ischemic heart disease, it is important to recognize that the results may not be generalizable to other populations with different medical or cultural conditions. Additionally, there could be a bias due to self-reporting, as there is a possibility that participants responded in a socially desirable manner.
